# A novel approach for engineering efficient nanofluids by radiolysis

**DOI:** 10.1038/s41598-022-14540-z

**Published:** 2022-06-24

**Authors:** M. Maaza, T. Khamliche, M. Akbari, N. Kana, N. Tandjigora, P. Beukes, A. Genu, K. Kaviyarasu, J. K.Cloete, M. Lekala, A. Gibaud, M. Henini

**Affiliations:** 1 UNESCO-UNISA Africa Chair in Nanosciences-Nanotechnology, College of Graduate Studies, University of South Africa, Muckleneuk Ridge, PO Box 392, Pretoria, South Africa; 2 Nanosciences African Network (NANOAFNET), Materials Research Dept., iThemba LABS-National Research Foundation of South Africa, 1 Old Faure Road, Somerset West, PO Box 722, Western Cape, 7129 South Africa; 3 Department of Physics, University of South Africa, Muckleneuk Ridge, PO Box 392, Pretoria, South Africa; 4 IMMM, UMR 6283 CNRS, University of Le Maine, 72085 Bd O. Messiaen, Le Mans Cedex 09, France; 5 Physics and Astronomy Department, Nottingham University, Nottingham, NG7 2RD7 UK

**Keywords:** Energy science and technology, Engineering, Materials science, Nanoscience and technology, Physics

## Abstract

This contribution reports for the first time the possibility of using radiolysis to engineer stable efficient nanofluids which exhibit an enhanced thermal conductivity. The validation was confirmed on Ag-H_2_O and Ag-C_2_H_6_O_2_ nanofluids fabricated via g-radiolysis within the mild dose range of 0.95 × 10^3^–2.45 × 10^3^ Gray. The enhanced thermal conductivity of Ag-H_2_O and Ag-C_2_H_6_O_2_ nanofluids, was found to be g-radiations dose dependent. In the latter case of Ag-C_2_H_6_O_2_ nanofluid, the relative enhancement in the temperature range of 25–50 °C was found to be 8.89%, 11.54%, 18.69%, 23.57% and 18.45% for D_1_ = 0.95 × 10^3^ Gray, D_2_ = 1.2 × 10^3^ Gray, D_3_ = 1.54 × 10^3^ Gray, D_4_ = 1.80 × 10^3^ Gray and D_5_ = 2.45 × 10^3^ Gray respectively. Yet not optimized, an enhancement of the effective thermal conductivity as much as 23.57% relatively to pure C_2_H_6_O_2_ was observed in stable Ag-C_2_H_6_O_2_ nanofluids. Equivalent results were obtained with Ag-H_2_O.

## Introduction

In line with the current fast rising demand of our ICT driven society, and in search for more efficient coolants in nanoelectronics so to dissipate effectively the generated heat within as well as the heat generated in the fast-growing market of data storage centers, nanofluids are considered as a viable technology response^1,2^. Yet, initially investigated as a novel class of coolants for heat removal in nuclear reactors^3,4^ and the automotive industry^5^, nanofluids pioneered by Choi et al.^6^ are being investigated extensively in addition to their potential applications in geothermal energy and biomedical sectors^7,8^.

**Figure 1:**
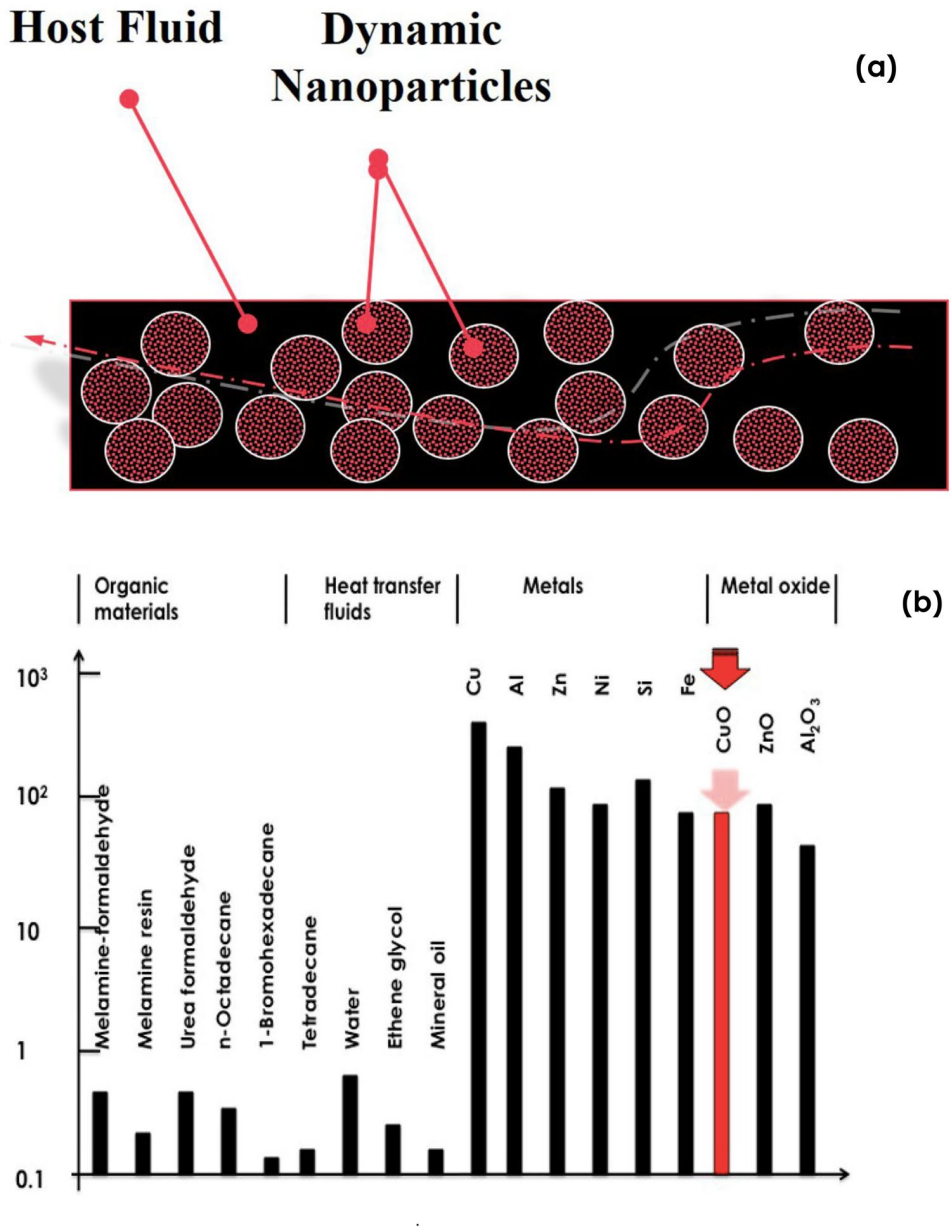
(**a**) Universal configuration of a nanofluid consisting of nanoscaled particles in suspension in a host standard fluid, (**b**) comparative scale of thermal conductivity of various materials: organic materials, standard heat transfer fluids, metals and their oxides.

As shown in Fig. 1a, nanofluids are a form of molecular fluids consisting of a uniform dispersion of nanoparticles in a traditional coolant host fluid such as H_2_O, oil or ethylene glycol (C_2_H_6_O_2_) amongst others. Figure 1b reports a comparison between the thermal conductivity of several organic materials, standard heat transfer fluids (water, ethylene glycol, mineral oil), metals and metal oxides. As one can notice, the thermal conductivity of standard heat transfer fluids is, inherently, lower than < 1 W m^−1^ K^−1^ at room temperature whilst that of metals and their corresponding oxides are 2–3 orders of magnitudes higher. Hence, the mixture of such metallic nanoparticles or their oxides in standard coolant host fluid in a form of a nano-suspension would induce a significant enhancement in the thermal conductivity of the nanofluid. While predicted and treated initially by Maxwell^9^, such an enhancement has been theoretically quantified by Batchelor and O’Brien in 1977^10^ and Hamilton, Grosser et al., as early as 1962^11^.

Indeed, such an enhancement of the thermal conductivity was confirmed experimentally in various nanofluids such as Al_2_O_3_-C_2_H_6_O_2_, TiO_2_-C_2_H_6_O_2_, CNTs- C_2_H_6_O_2_, CuO-H_2_O, ZnO-H_2_O, Ag-H_2_O, CNTs-H_2_O^12^. This reproducible thermal conductivity enhancement was reported varying from 7 to 18% relatively to that of the host medium of C_2_H_6_O_2_ or H_2_O. Recently, Mbambo et al. reported an enhancement of about 33% in a multi-components nanosystem of Ag or Au grafted Graphene/C_2_H_6_O_2_ based nanofluids^16,17^.

**Figure 2:**
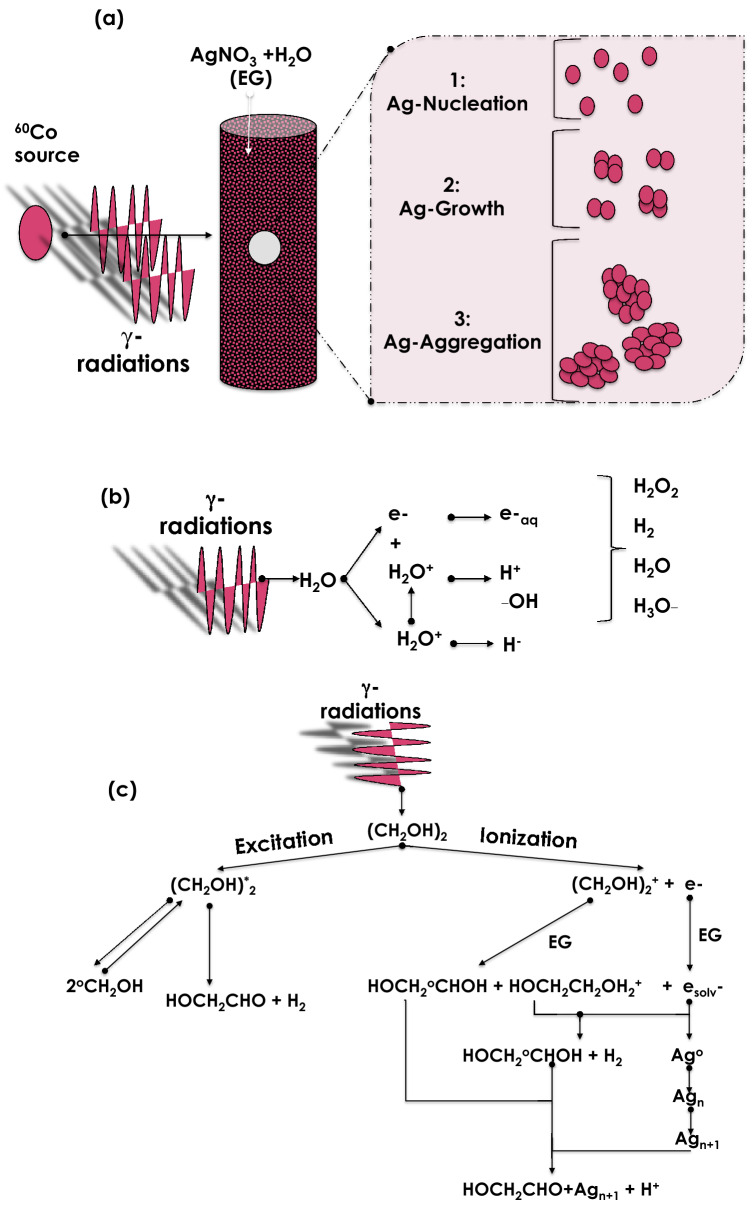
(**a**) Principle of the formation of nanofluids with gamma radiolysis of Ag nanoparticles dispersed in either H_2_O or C_2_H_6_O_2_, (**b**) major scavenging intermediates species including H_2_O^+^, H^+^, ^_^OH, H^−^, e^−^, and e^−^_aq_ involved in the mechanism of H_2_O radiolysis, (**c**) major intermediates species and chemical reactions involved in the mechanism of C_2_H_6_O_2_ radiolysis and the formation of colloidal suspensions of nano-scaled Ag particles.

For the synthesis of stable nanofluids, two major approaches are followed so far; namely single and double steps approaches. While in the double steps approach, the nanoparticles are produced by various nano-synthesis physical or chemical methods and then dispersed in the host thermal host fluid with the possibly an additional surfactant molecular agent to minimize their agglomeration. Henceforth, preventing their Otswald-ripening equivalent agglomeration. In the single step version, however, the nanoparticles are directly generated within the host fluid itself. The single step methodologies comprise the followings: (1) Evaporation^6^, (2) Microwave^19^, (3) Pulsed laser ablation in liquid solution^20^, (4) Electric arc-discharge^12^, and (5) Sonochemistry^21^. Yet the subject of radiolysis is well established in radiochemistry and radiobiology^22^, this contribution reports on for the first time and validates the possibility of using radiolysis for the synthesis of stable efficient nanofluids which exhibit enhanced thermal conductivity. Hence, the originality and novelty of this contribution lies within the first time usage of radiolysis as a mean of nanofluids’ synthesis. The advantage of engineering nanofluids by radiolysis is its cost effectiveness and potential mass production inherent characteristics. Figure 2a summarizes the principle of the formation of nanofluids with gamma radiolysis of Ag nanoparticles dispersed in either H_2_O or C_2_H_6_O_2_. The corresponding radiolysis chemical reactions are summarized in Fig. 2b, c and will be discussed further later in the manuscript.

Relatively to each and all nanofluids synthesis methodologies, and as per sustained by the reported experimental results, Radiolysis seems to have the advantage of process’ simplicity, upscaling and mass production as well as relatively a minimal energy input in addition to the no requirement for vacuum.

As per the published scientific and engineering literature, the targeted major applications of nanofluids by the international community are: (1) Heat transfer, heat removal and cooling applications, (2) Automotives applications, (3) Electronic applications and cooling of data storage centres, (4) Biomedical technologies, (4) Detergents, (6) Ultra deep drilling, and (7) Geothermal applications^23^.

This contribution is motivated by the above mentioned demand for developing nanofluids by less energy intensive and/or cost effective single step fabrication processes. As, it will presented later, the enhanced thermal conductivity of Ag-H_2_O and Ag-C_2_H_6_O_2_ nanofluids by radiolysis, was found to be dose dependent. More precisely for Ag-C_2_H_6_O_2_ nanofluid, the enhancement relatively to the host standard fluid of C_2_H_6_O_2_ in the temperature range of 25–50 °C was found to be 8.85%, 11.9%, 18.7%, 23.7% and 18.3% for D_1_ = 0.95 × 10^3^ Gray, D_2_ = 1.2 × 10^3^ Gray, D_3_ = 1.54 × 10^3^ Gray, D_4_ = 1.80 × 10^3^ Gray and D_5_ = 2.45 × 10^3^ Gray respectively. Yet not optimized, a significant enhancement of the effective thermal conductivity as much as 23.7% relatively to pure C_2_H_6_O_2_ was observed in stable Ag-C_2_H_6_O_2_ nanofluids. Similar results were obtained with the Ag-H_2_O nanofluids.

## Experiments, results and discussions

### Synthesis and methodology

For the samples preparation, silver nitrate (AgNO_3_, Merck, Germany) was used as the starting source of silver for the radiolytic-induced reduction of Ag^+^ to Ag^0^. De-ionized H_2_O and standard purity C_2_H_6_O_2_ solutions containing 40 mM AgNO_3_ were prepared. After deaeration by bubbling with nitrogen gas, the solutions were irradiated by gamma rays emitted by a standard panoramic 1MCi Co_-60_ source in a regular configuration as schematically represented in Fig. 2a. The standard irradiations were carried out at various gamma irradiation doses of D_1_ = 0.95, D_2_ = 1.25, D_3_ = 1.54, D_4_ = 1.80 and D_5_ = 2.45 × 10^3^ Gray and at a dose rate of 10.0 10^3^ Gy/h for each of the prepared 40 mM AgNO_3_ in H_2_O and in C_2_H_6_O_2_ solutions. These doses were chosen based on the published literature^31–33^. More precisely, such a set of doses allows the synthesis of homogeneous nano-scaled Ag colloidal suspensions^31–33^, with a relative stability of months^34^. Figure S1 summarizes the experimental g-radiolysis methodology.

### Materials and nanofluid characterization

The morphology, size distribution and crystallographic structure of the Ag nanoparticles within the Ag-H_2_O and Ag-C_2_H_6_O_2_ nanofluids were studied by using a JEOL JEM 2010F Transmission Electron microscopy unit. The optical Plasmonic investigations were conducted on an Ocean Optics UV–VIS-NIR spectroscopy unit within the spectral range of 200–500 nm. The thermal conductivity of the engineered nanofluids was investigated by the standard transient hot-wire technique^24^ within the temperature range of 25–50 °C. It is to be highlighted that such a limitation to such a temperature range of 25–50 °C was imposed by 2 major factors; (1) generally, the thermal conductivity of nanofluids by hot-wire approach are reported within such a temperature range^1,12–17,20^ and (2) the stability of the thermal conductivity measurements seems significant within such a thermal range on the used system. As established, the accuracy of this hot wire approach (order + 0.2%) and precision (order 0.02%) have been obtained as a result of the application of modern electronic instruments of a superior quality^24,42,50^.

### Morphological and size distribution investigations

**Figure 3:**
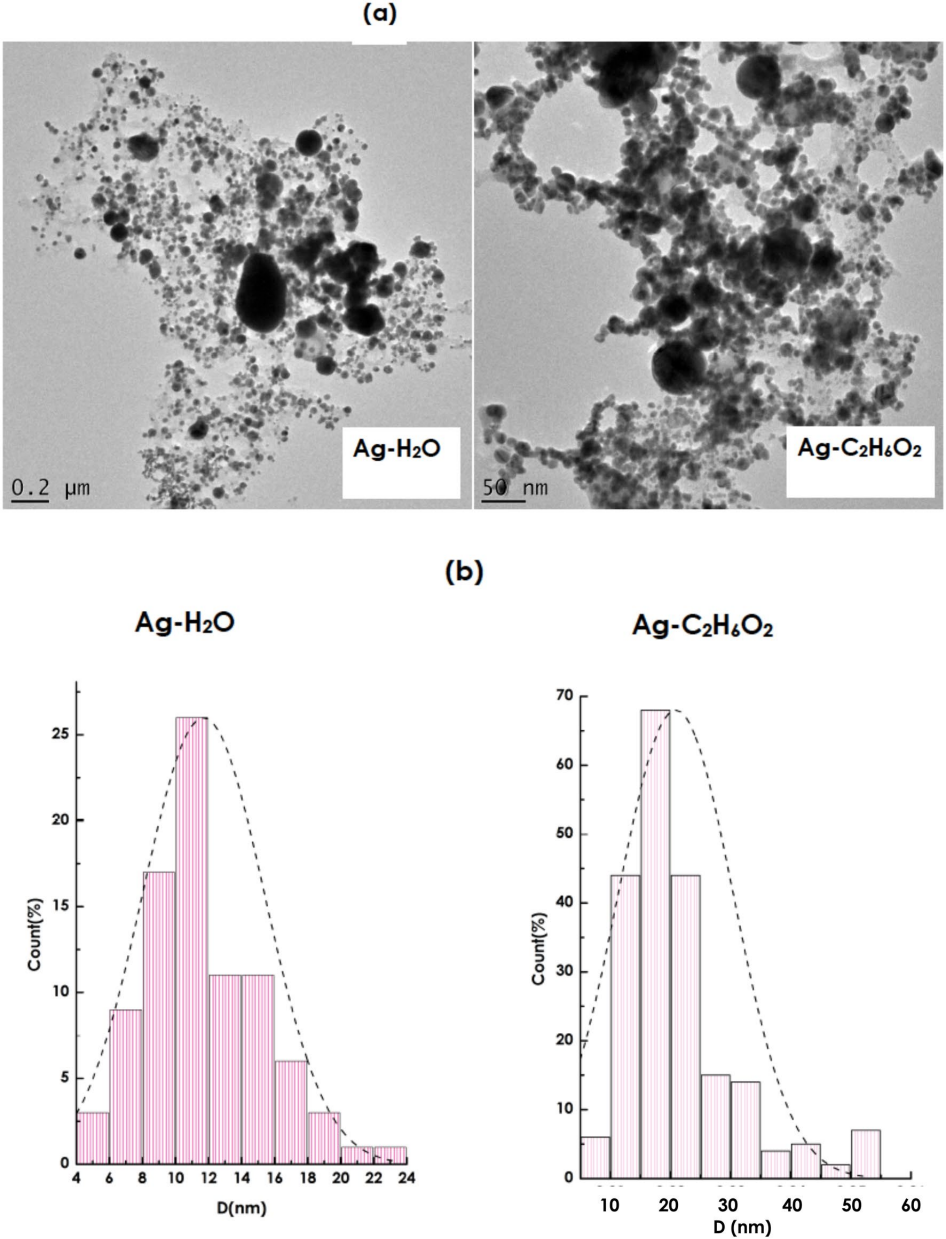
(**a**) Transmission electron microscopy images of the Ag nanoparticles in H_2_O and C_2_H_6_O_2_, (**b**) size distribution of the synthesized Ag nanoparticles by radiolysis in H_2_O and C_2_H_6_O_2_.

Figure 3a reports a typical Transmission Electron Microscopy of the radiolized Ag nanoparticles in H_2_O and C_2_H_6_O_2_ based nanofluids following the g- irradiation at a dose of 1.80 × 10^3^ Gray. The Ag nanoparticles seem to be quasi-spherical in shape with a likely-gaussian size distributions (Fig. 3b) in both H_2_O and C_2_H_6_O_2_. The Gaussian-like distributions are centred at about 12 nm and 27 nm in H_2_O and C_2_H_6_O_2_ respectively. These observations confirm the effectiveness of radiolysis for engineering nanofluids in standard heat transfer media i.e. H_2_O and C_2_H_6_O_2_.In terms of stability, it is well established that colloidal nanoparticles in general and Ag nanoparticles especially, made by radiolysis in H_2_O are very stable^35–38,40^. Such a long term stability is attributed to the electric dipole of the water molecules which is of 1.84 Debyes. Because of the superior electric dipole of C_2_H_6_O_2_ molecules (2.75 Debyes), which is nearly twice of that H_2_O molecules (1.84 Debyes) it could be concluded safely that the Ag–C_2_H_6_O_2_ nanofluids would be more stable than the Ag-H_2_O nanofluids.

### Mechanism of radiolysis and formation of Ag nanoparticles

Since its inception, the mechanism of radiolysis of H_2_O has been extensively documented. The formation of nanoscaled particles in suspension in H_2_O is bound to several scavenging intermediates species including H_2_O^+^, H^+^, –OH, H^−^, e^−^, and e^−^_aq_. The whole mechanism can be summarized as schematically described in Fig. 2b. By contrast, the radiolysis of C_2_H_6_O_2_ has not been documented as much as the H_2_O’s one.

Within the g-photolysis of C_2_H_6_O_2_, the pivotal decomposition of CH_2_OHCH_2_O was postulated by Van der Linde and Von Sontag as early as 1971^25^. This was followed by radiolysis of aqueous solutions of several monobasic alcohols in view of validating such a decomposition. In the case of glycols, studies reported on the radiolytic transformations in aqueous solutions in the presence and absence of Oxygen. It was found that the yield of the produced glycolic aldehyde was concentration and purity dependent^26–30^.

Within the same family of Ethylene Glycol, Mostafavi et al. have investigated thoroughly both in steady regime and time resolved, the radiolysis of methanol (CH_3_OH). It was concluded that the yields of solvated electrons (e_solv._^−^) and radical scavenging rates were correlated^31^. Likewise, the presence of Ag ions or charged clusters which scavenge both solvated electrons (e_solv_^−.^) and ^·^CH_2_OH/CH_3_O^·^ radicals were observed allowing the identification of the full scheme of radiolytic mechanism of Methanol with the yields of the various potential pathways^32^.

Applying similar procedure, Soroushian, Mostafavi et al. have investigated the radiolysis of C_2_H_6_O_2_ as well as the radiolysis of Ag ion within it^33^. Based on various transient studies of solvated electrons (e_solv._^−^) in the nanosecond^34–37^ and the femtosecond^38^ regimes as well as the model of metal clusters growth in liquids of Henglein^39^; and Belloni et al.^40^, Soroushian et al. have identified not only the various radiolytic mechanisms in the g-photolysis of C_2_H_6_O_2_ but also the corresponding scavenging yields and the radiolytic species as well as the rate constants of Ag ions. Accordingly, the corresponding radiolytic mechanism is described as per the schematic description of Fig. 2c based on the following reactions^33,41^:

1 $${\text{e}}^{ - } \to {\text{e}}_{{{\text{solv}}.}}^{ - }$$

2 $$\left( {{\text{CH}}_{{2}} {\text{OH}}} \right)_{2}^{ + \cdot } + \left( {{\text{CH}}_{{2}} {\text{OH}}} \right)_{{2}} \to {\text{HOH}}_{{2}} {\text{CC}}^{ \cdot } {\text{HOHCH}}_{{2}} {\text{OH}}^{ \cdot } + {\text{HOH}}_{{2}} {\text{CCH}}_{{2}} {\text{OH}}_{2}^{ + }$$

3 $$\left( {{\text{CH}}_{{2}} {\text{OH}}} \right)_{2}^{*} \to {\text{HOH}}_{{2}} {\text{CC}}^{ \cdot } {\text{HO}} + {\text{2H}}^{ \cdot }$$

4 $$\left( {{\text{CH}}_{{2}} {\text{OH}}} \right)_{2}^{*} \to {\text{HOH}}_{{2}} {\text{CCHO}} + {\text{H}}_{{2}}$$

5 $$\left( {{\text{CH}}_{{2}} {\text{OH}}} \right)_{2}^{*} \to {2} {^{\cdot}\text{CH}}_{{2}} {\text{OH }} +$$

6 $$\left( {{\text{CH}}_{{2}} {\text{OH}}} \right)_{{2}} + {\text{H}}^{ \cdot } \to {\text{HOH}}_{{2}} {\text{CC}}^{ \cdot } {\text{HOH}} + {\text{H}}_{{2}}$$

7 $${\text{e}}_{{{\text{solv}}.}}^{ - } + {\text{ HOH}}_{{2}} {\text{CCH}}_{{2}} {\text{OH}}_{2}^{ + } \to \left( {{\text{CH}}_{{2}} {\text{OH}}} \right)_{{2}} + {\text{ H}}^{ \cdot } \to {\text{HOH}}_{{2}} {\text{CC}}^{ \cdot } {\text{HOH }} + {\text{ H}}_{{2}}$$

8 $${\text{2HOH}}_{{2}} {\text{CC}}^{ \cdot } {\text{HOH}} \to \left( {{\text{CH}}_{{2}} {\text{OH}}} \right)_{{2}} + {\text{ HOH}}_{{2}} {\text{CC}}^{ \cdot } {\text{HO}}$$

9 $${2} {^{ \cdot }\text{CH}}_{{2}} {\text{OH}} + {\text{H}}^{ \cdot } \to \left( {{\text{CH}}_{{2}} {\text{OH}}} \right)_{{2}}$$

10 $${\text{e}}_{{{\text{solv}}.}}^{ - } + {\text{e}}_{{{\text{solv}}.}}^{ - } \to {\text{H}}_{{2}} + .{\text{ 2HOH}}_{{2}} {\text{CCH}}_{{2}} {\text{O}}^{ - }$$

11 $${\text{e}}_{{{\text{solv}}.}}^{ - } + {\text{HOH}}_{{2}} {\text{CCHO}}^{ - } \to {\text{HOH}}_{{2}} {\text{CCHO}}^{ - \cdot }$$

Following this set of reaction, the silver clusters would be produced by the coalescence of silver atoms arising from the scavenging reaction of solvated electrons and possibly H^·^ atoms, as much as the H^•^ atoms are not scavenged by C_2_H_6_O_2_. Their formation is governed as follows:

12 $${\text{e}}_{{{\text{solv}}.}}^{ - } + {\text{Ag}}^{ + .} \to {\text{Ag}}^{0}$$

13 $${\text{H}}^{ \cdot } + {\text{Ag}}^{ + \cdot } \to {\text{H}}^{ + } + {\text{Ag}}^{0}$$

14 $${\text{Ag}}^{0 \cdot } + {\text{Ag}}^{ + \cdot } \to {\text{Ag}}_{2}^{ + }$$

15 $${\text{Ag}}^{{{\text{x}} + \cdot }} + {\text{Ag}}^{{{\text{y}} + \cdot }} \to {\text{Ag}}^{{{\text{(x}} + {\text{y}}) + }}$$

### Crystallograhic and structural investigations

**Figure 4:**
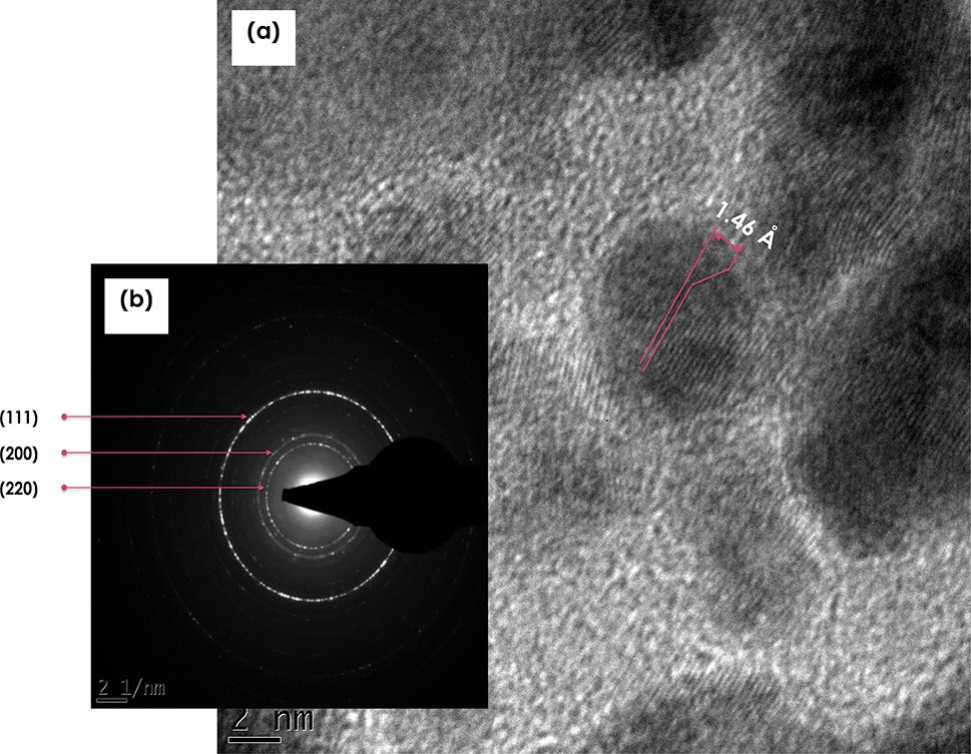
(**a**) Transmission electron microscopy images of the Ag nanoparticles in H_2_O and C_2_H_6_O_2_, (**b**) electron diffraction pattern of the Ag nanoparticles in Ag-C_2_H_6_O_2_ radiolized at D_4_ = 1.80 × 10^3^ Gray.

Figure 4a reports the High Resolution Transmission Electron Microscopy (HRTEM) image of the 1.80 × 10^3^ Gray radiolized Ag nanoparticles in C_2_H_6_O_2_. Accordingly, there are both amorphous and polycrystalline Ag nanoparticles. Those crystalized seem to exhibit a preferred crystal orientation with an inter-reticular d_hkl_ distance of 1.46 Å corresponding, a priori, to the Ag (220) reticular plans. This later is in agreement with the Selected Area Electron Diffraction (SAED) pattern of Fig. 4b whereby the electron diffraction ring (220) is, relatively, the most intense suggesting a (220) preferential textured orientation.

**Figure 5:**
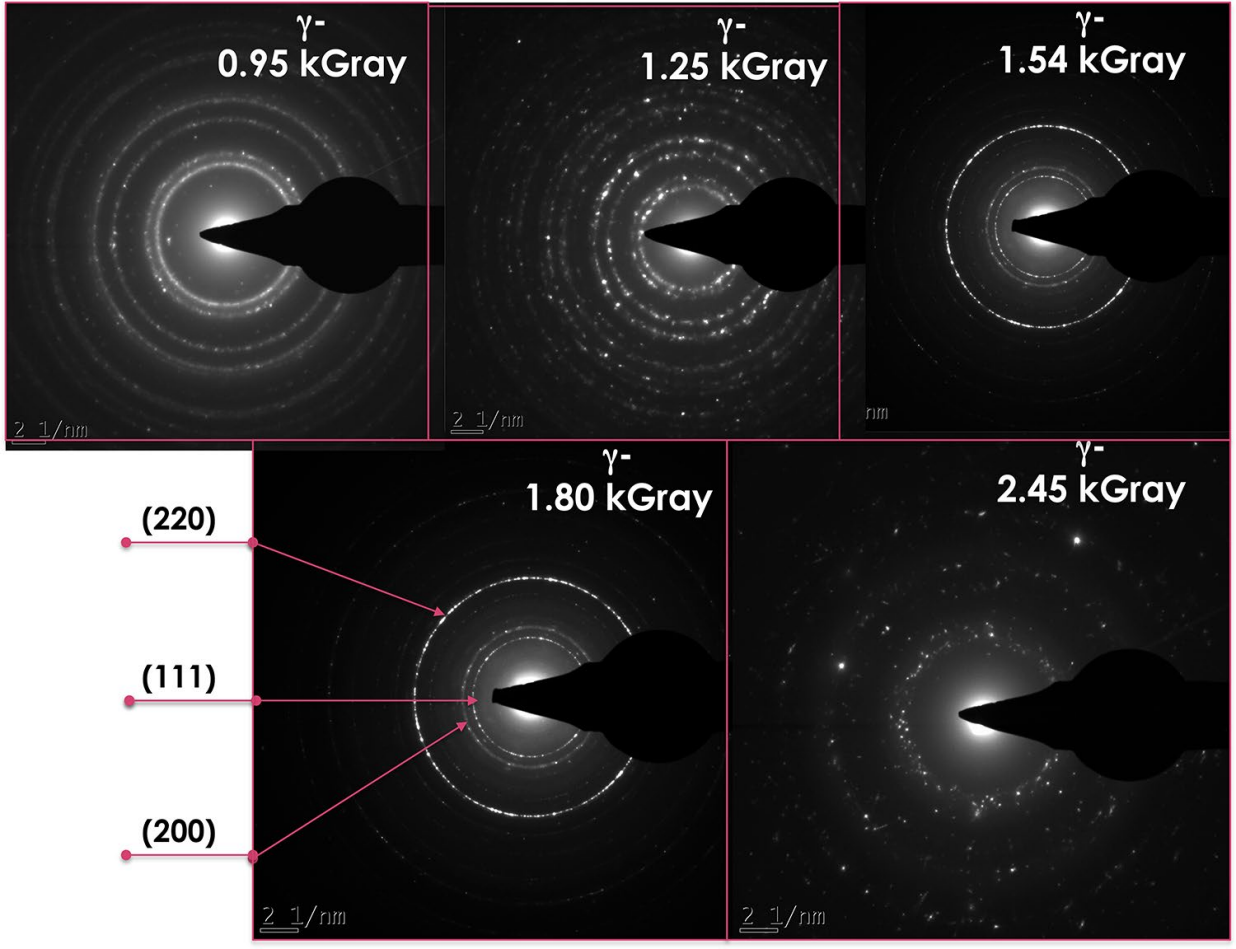
Electron diffraction patterns of the Ag nanoparticles in Ag-C_2_H_6_O_2_ radiolized at the various doses of D_1_ = 0.9510^3^, D_2_ = 1.25 × 10^3^, D_3_ = 1.54 × 10^3^, D_4_ = 1.80 × 10^3^ and D_5_ = 2.45 × 10^3^ Gray.

It is however worth mentioning that the degree of crystallinity of the Ag nanoparticles seems to be dose dependant. Figure 5 reports the SAED patterns of the Ag nanoparticles radiolized in C_2_H_6_O_2_ at various gamma irradiation doses of D_1_ = 0.95 × 10^3^, D_2_ = 1.25 × 10^3^, D_3_ = 1.54 × 10^3^, D_4_ = 1.80 × 10^3^ and D_5_ = 2.45 × 10^3^ Gray. The samples radiolized at D_1_ = 0.95 × 10^3^ and D_2_ = 1.25 × 10^3^ Gray exhibit both amorphous and polycrystalline nanoparticles while those radiolized at D_3_ = 1.54 × 10^3^ and D_4_ = 1.80 × 10^3^ Gray display equally a preferred texture with a relatively sharp (220) orientation. The highest radiolized sample of D_5_ = 2.45 × 10^3^ Gray exhibits crystallized nanoparticles with a broad variety of crystallographic orientations.

### Optical studies and plasmonic response

**Figure 6:**
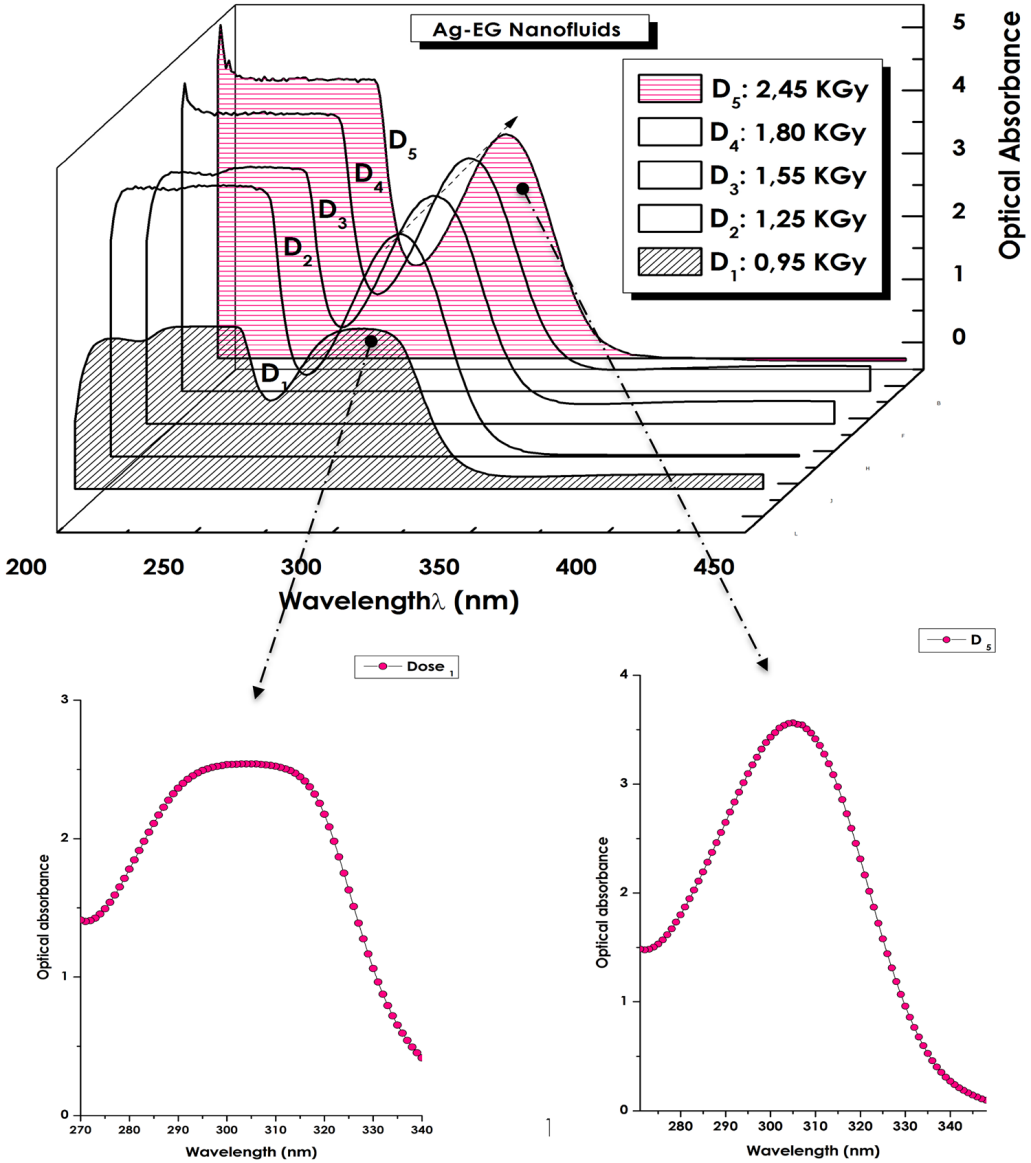
UV–VIS-NIR absorbance of the various Ag-C_2_H_6_O_2_ nanofluids radiolized at D_1_ = 0.9510^3^, D_2_ = 1.25 × 10^3^, D_3_ = 1.54 × 10^3^, D_4_ = 1.8010^3^ and D_5_ = 2.45 × 10^3^ Gray.

Figure 6 displays the optical absorbance within the spectral range of 200–450 nm of the various Ag–C_2_H_6_O_2_ nanofluids radiolized at various gamma irradiation doses of D_1_ = 0.95 × 10^3^, D_2_ = 1.25 × 10^3^, D_3_ = 1.54 × 10^3^, D_4_ = 1.80 × 10^3^ and D_5_ = 2.45 × 10^3^ Gray. One can notice the intrinsic plasmonic peak confirming the formation and the homogeneous colloidal dispersion of Ag nanoparticles within the host matrix of C_2_H_6_O_2_. For the nanofluids radiolized at D_2_ = 1.25 × 10^3^ Gray and above, the width at half maximum of the Ag plasmonic peak is relatively constant (Dl_1/2_ ~ 31.8 nm) for such samples suggesting the homogeneity of the average size of the Ag nanoparticles within the host matrix of C_2_H_6_O_2_. By contrast, the situation for the nanofluid radiolized at the lowest dose i.e. D_1_ = 0.95 × 10^3^ Gray is relatively different. Firstly, the plasmonic peak’s width at half maximum is broader (Dl_1/2_ ~ 77.2 nm) and seems consisting of a superposition/juxtaposition of several peaks. At a first approximation, not less than 4 Lorentzian profiles are required for its full simulation. Henceforth and, at first glance, one could associate each of the profiles as related to a specific class “i” of Ag nanoparticles with an average size <Ø_i_> . In view of the lower intensity of this plasmon peak and its relatively large width at half maximum, a priori, the D_1_ = 0.95 × 10^3^ Gray fluence could be the threshold gamma radiation from which the Ag nanoparticles start to form. Likely, this later seems corresponding to stage 2 labelled as Ag-growth as reported in the schematic description of Fig. 2a.

### Thermal conductivity enhancement studies

**Figure 7:**
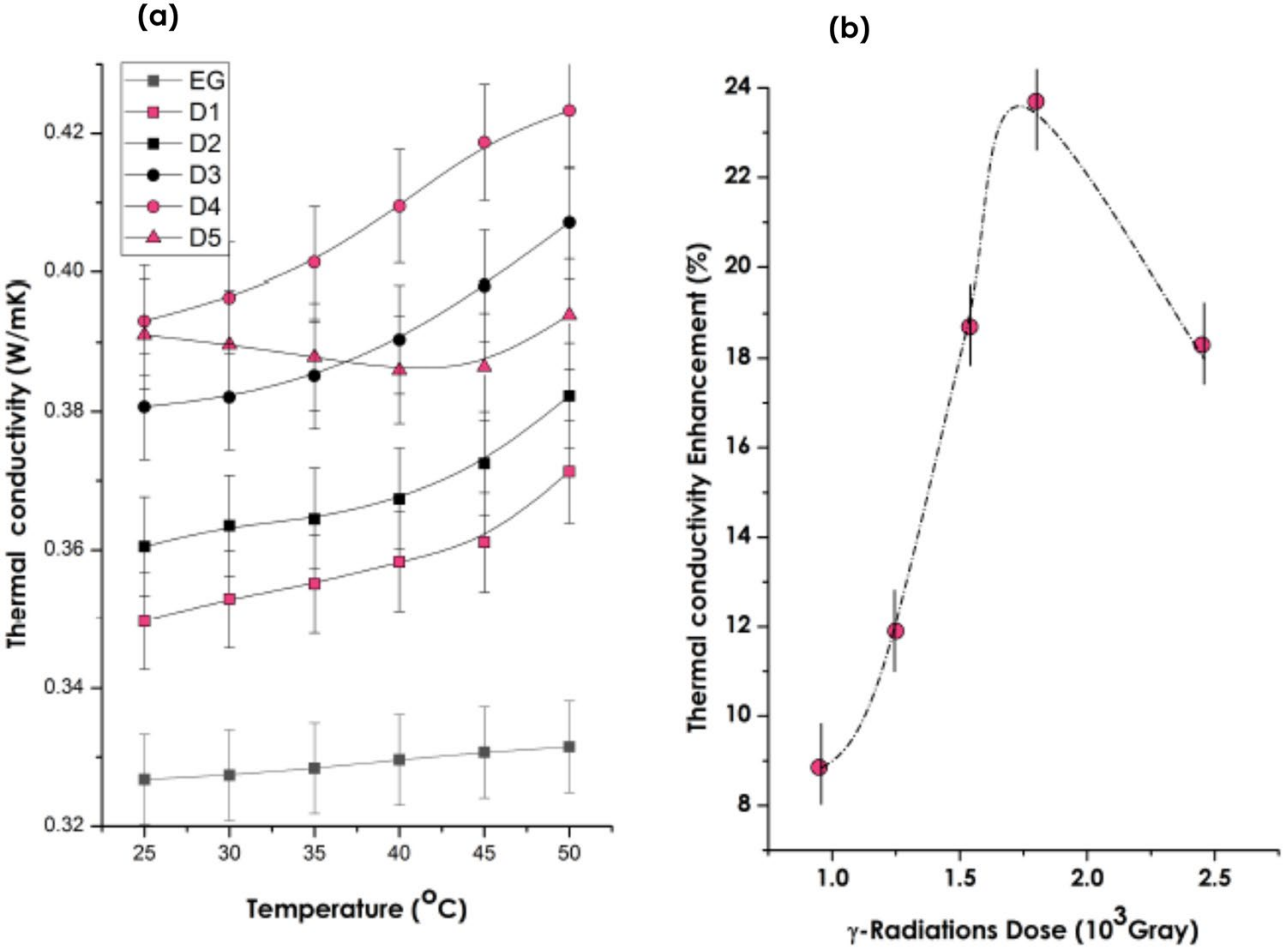
(**a**) Thermal conductivity of the various Ag-C_2_H_6_O_2_ nanofluids radiolized at D_1_ = 0.9510^3^, D_2_ = 1.25 × 10^3^, D_3_ = 1.54 × 10^3^, D_4_ = 1.8010^3^ and D_5_ = 2.45 × 10^3^ Gray, (**b**) average thermal conductivity measured within the 25–50 °C range versus the g-radiations dose.

As it was mentioned previously, the thermal conductivity measurements were carried out on a hot-wire unit schematically described in Fig. S2. Figure 7a reports the thermal conductivity of the various Ag-C_2_H_6_O_2_ nanofluids synthesized at various doses as well as the thermal conductivity of pure C_2_H_6_O_2_ (as a reference) in the standard temperature range of 25–50 °C. In general and relatively to pure C_2_H_6_O_2,_ there is a net enhancement of the thermal conductivity of the various nanofluids relatively to pure C_2_H_6_O_2_. As previously mentioned, the accuracy of this hot wire approach (order + 0.2%) and its precision (order 0.02%) are attained as a result of the usage of advanced electronic instruments^24,42^.

The average thermal conductivity within such a temperature range is 0.3581, 0.3684, 0.3906, 0.4071 and 0.3892 W/m K for the nanofluids radiolized at D_1_ = 0.95 × 10^3^ Gray, D_2_ = 1.2 × 10^3^ Gray, D_3_ = 1.54 × 10^3^ Gray, D_4_ = 1.80 × 10^3^ Gray and D_5_ = 2.45 × 10^3^ Gray respectively. The measured average thermal conductivity of the host fluid i.e. C_2_H_6_O_2_ is 0.3290 W/m K. Likewise, and excluding the Ag-C_2_H_6_O_2_ nanofluid synthesized at the highest dose (D_5_ = 2.45 × 10^3^ Gray), the thermal conductivity increases regularly with temperature in the considered limited temperature range of 25–50 °C. As summarized in Fig. 7b, this translates into an increase of the relative average thermal conductivity ϑ_1_ from 8.89%, 11.54%, 18.69%, 23.57% and 18.45% for D_1_ = 0.95 × 10^3^ Gray, D_2_ = 1.2 × 10^3^ Gray, D_3_ = 1.54 × 10^3^ Gray, D_4_ = 1.80 × 10^3^ Gray and D_5_ = 2.45 × 10^3^ Gray respectively.

To sustain and conclude on the enhancement of the thermal conductivity and its reproducibility, additional measurements were carried out. More precisely, for each Dose D_i_, 5 measurements within the thermal temperature range of 25–50 °C were performed (each time, 6 values corresponding to 25, 30, 35, 40, 45 and 50 °C) and the relative average enhancement ϑ_i_ (%) was derived. The same procedure was repeated 5 times. The corresponding results of this lengthy exprimental section is summarized in Table 1. As one can notice, the relative enhancement ϑ_i_ (%) for each dose is relatively constant within the error bar of uncertainty.

**Table 1:** Summary of the relative average enhancement ϑ_i_ (%) for each dose. The average was made over 5 values of the thermal conductivity at 25, 30, 35, 40, 45 and 50 °C.

	ϑ_1_ (%)	ϑ_2_ (%)	ϑ_3_ (%)	ϑ_4_ (%)	ϑ_5_ (%)
D_1_	9.167	9.251	8.529	8.448	9.059
D_2_	11.951	11.553	11.469	11.304	11.415
D_3_	18.710	18.403	18.930	19.041	18.376
D_4_	23.670	23.480	23.974	23.311	23.423
D_5_	18.291	18.042	18.568	18.653	18.710

While the increase of the relative thermal conductivity of the Ag-C_2_H_6_O_2_ nanofluid versus the gamma radiation dose is expected, its decay for the highest dose D_5_ is unexpected. The increase of the dose induces a larger formation of Ag nanoparticles and hence their volume concentration which would be translated in an increase of the thermal conductivity and hence the observed increase up to D_4_. As the decrease at the highest dose D_5_ can not be explained for the moment. It is intended to carry out more precise studies within the range of 1.80 × 10^3^–2.45 × 10^3^ Gray in view of elucidating such an unexpected decrease.

**Figure 8:**
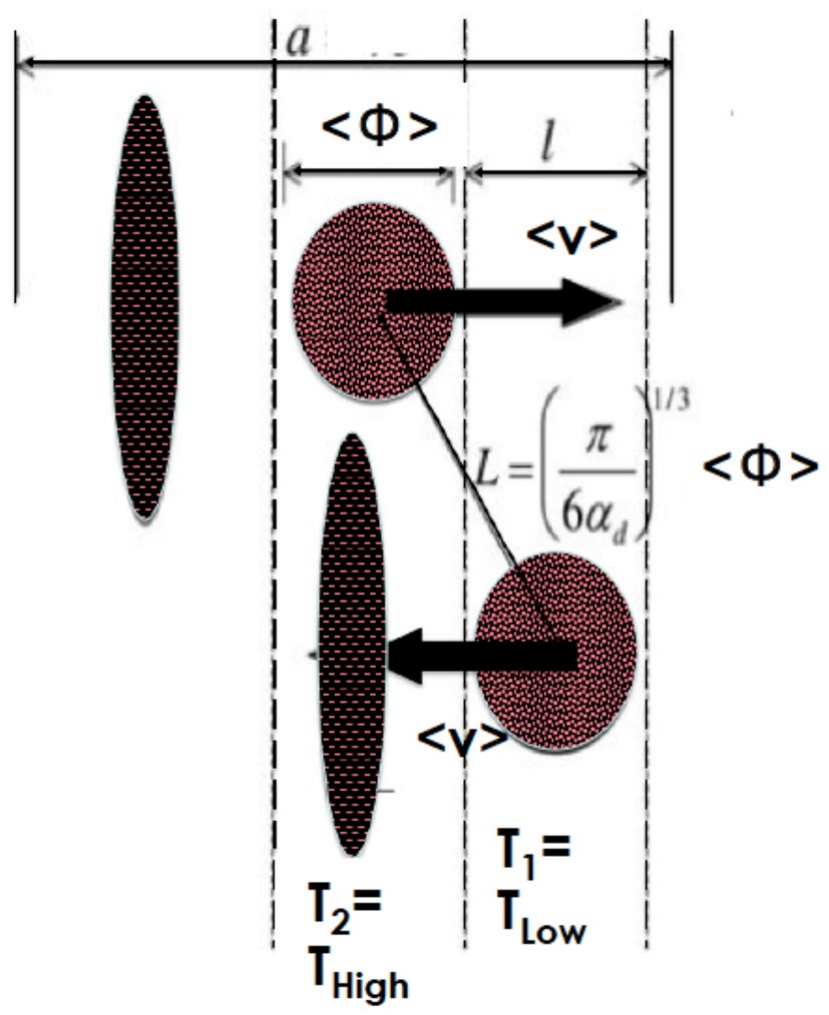
Schematic description and derivation of the Brownian component of the thermal conductivity of nanoparticles in a host fluid due to their Brownian motion^43–45^.

As mentioned previously, the increase of the thermal conductivity with temperature as shown in Fig. 7a is generally accepted as due to the Brownian motion. It is accepted by the community as a whole that the effective thermal conductivity, *k*_*eff*_, of a nonofluid consists of 2 major components; the static *k*_*stat*_ and the Brownian *k*_*Brow*_ as *k*_*eff*_ = *k*_*Stat*_ + *k*_*Brow*_ with *k*_*Stat*_ given by Maxwell’s approximation as (9):

16 $$k_{Stat} = \frac{{k_{c} \left( {1 + 3\left[ {\left( {\frac{{k_{d} }}{{k_{c} }} - 1} \right)\alpha_{d} } \right]} \right.}}{{\left( {\frac{{k_{d} }}{{k_{c} }} + 2} \right) - \left( {\frac{{k_{d} }}{{k_{c} }} - 1} \right)\alpha_{d} }}$$

with *α*_*d*_, *k*_*c*_ and *k*_*d*_ are the nanoparticles volume fraction, the thermal conductivity of the fluid carrier and that of the nanoparticles respectively. The Brownian component is driven by the temperature’s induced translational motion of the nanoparticles as schematically represented in Fig. 8. The average translational time-averaged speed of the nanoparticles *ν*_*d*_ has been deduced by Probstein^45^ as:

17 $$\left\langle {\nu_{d} } \right\rangle = \sqrt {18k_{B} T/\pi \rho_{d} \left\langle \emptyset \right\rangle^{3} }$$

where *ρ*_*d*_, $\langle \emptyset \rangle$, *k*_*B*_ are the nanoparticles’ density, their average diameter and the Boltzmann’s constant. At room temperature, *ν*_*d*_ is of the order of 1.63 m/s, 5.15 × 10^–2^ m/s and 1.63 × 10^–3^ m/s for nanoparticles with $\langle \emptyset \rangle = 10,\,100,\,\,{\text{and}}\,1000\,{\text{nm}}$ respectively. Accordingly, the Brownian motion can not be neglected for small nanoparticles especially those with diameter within the range of 10 nm. The heat transported by the nanoparticles from a hot to a cold section can be derived defining *p* as the probability for a particle to travel along any direction, and assuming that each of the two particle cells are in thermal equilibrium at temperatures of *T*_1_ and *T*_2_, respectively, these particles moving to neighboring cells (Fig. 8) will carry energy across the interface as^43^:

18 $$Q_{net} = \frac{\Delta Q}{{A\Delta t}} \approx \frac{{(pNm_{d} )C_{v} (T_{1} - T_{2} )}}{A\Delta t} = - \frac{{pNm_{d} C_{v} \left\langle {\nu_{d} } \right\rangle \left( {\frac{\Delta T}{l}} \right)l}}{{A\left\langle {\nu_{d} } \right\rangle dt}}$$

With $\left( {\frac{\Delta T}{l}} \right)\sim \,\nabla T$, *N* is the total particle number in a cell as approximated in Fig. 8, *m*_*d*_ the nanoparticles’ mass, *ρ*_*d*_ their density, and *C*_*ν*_ their specific heat, *V*_*d*_ is the particle volume, *m*_*d*_ = *ρ*_*d*_*V*_*d*_, while *A* is the cross-sectional area of the system normal to the propagation x-direction, $A\langle \nu_{d} \rangle \Delta t = V$, $NV_{d} /V = \alpha_{d}$, One obtains^43,44^:

19 $$Q_{net} = - p\alpha_{d} \rho_{d} C_{v} \left\langle {\nu_{d} } \right\rangle \left\langle \emptyset \right\rangle \nabla T$$

20 $$Q_{net} = - k_{Brow} \nabla T$$

21 $$k_{Brow} = \sqrt {18k_{B} /\pi } p\alpha_{d} \sqrt {\rho_{d} } C_{v} \sqrt {T/\left\langle \emptyset \right\rangle }$$

According to expression (6), on can notice that the Brownian component of the thermal conductivity *k*_*Brow*_ becomes prominent for small particles ($k_{Brow} \propto l/\langle \emptyset \rangle$) and explains its observed increase with temperature in Fig. 7a ($k_{Brow} \propto \sqrt T$) for all samples radiolized at D_1_, D_2_, D_3_, D_4_ but not D_5_.

This last specific inconsistency could be due to the fact that the corresponding sample, i. e. the one radiolized with the highest g-dose of D_5_ = 2.45 × 10^3^ Gray, is likely to be the most concentrated. If so, the Brownian motion of the nanoparticles would favour their local agglomeration. In this case, while the heat transfer may be effective locally, it becomes ineffective from an agglomerate to a neighbouring agglomerate. If so, this would explain the decrease of thermal conductivity of the Ag-C_2_H_6_O_2_ nanofluid synthesized at D_5_ = 2.45 × 10^3^ Gray. To shed-light further on this observed result, and instead of speculating, a thorough and comprehensive investigations will be conducted out on nanofluids to be synthesized within the g-irradiation dose of 2.30 × 10^3^ and 2.50 × 10^3^ Gray and their thermal conductivity will be performed.

Also, it is worth noting that the highest thermal conductivity is exhibited by the nanofluids radiolysed at D_3_ and D_4_. In both cases, and as sustained by Fig. 5, the corresponding Ag nanoparticles present a relatively high crystallographic texture; (220). This seems indicating that the thermal conductivity of nanofluids is likely to be nanoparticles’atomic order dependent. As established in condensed matter^46^, heat propagation in crystals is, mainly carried by phonons, which scatter with each other, resulting in resistance. Therefore, the standard Phonon Gas Model (PGM) has been widely used to explore thermal conductivity in crystalline solids, in which phonons are treated as analogous to particles^47^. However, in structurally disordered media such as amorphous materials, the thermal behavior is quite different^48^ as the periodicity is an inherent requirement for defining phonons. The thermal conductivity in amorphous media was found to consist of 3 predominant components, named propagons, diffusons, and locons whereby Propagons are delocalized heat carriers with a rather identifiable wavevector in the low frequency range as summarized in the Allen and Feldman’ model^49^. It is, however premature, in this current study to conclude on the role of the crystallographic (220) texture on the thermal conductivity of the Ag nanoparticles. Henceforth, it is projected to conduct such an investigation as a foresight study.

## Conclusions

This contribution validated the possibility of engineering Ag-H_2_O and Ag-C_2_H_6_O_2_ based nanofluids by g-radiolysis within the dose range of 0.95 × 10^3^–2.45 × 10^3^ Gray. Such nanofluids exhibited a significant enhancement of the thermal conductivity which was found to be dose dependent. More precisely, In the case of Ag-C_2_H_6_O_2_ nanofluids, the relative enhancement in the temperature range of 25–50 °C was found to be from 8.89%, 11.54%, 18.69%, 23.57% and 18.45% for D_1_ = 0.95 × 10^3^ Gray, D_2_ = 1.210^3^ Gray, D_3_ = 1.54 × 10^3^ Gray, D_4_ = 1.80 × 10^3^ Gray and D_5_ = 2.45 × 10^3^ Gray respectively. Yet not optimized, the registered maximum of the enhancement of the thermal conductivity was as high as 23.57%. In addition, yet in a limited temperature range of 25–50 °C, the thermal conductivity enhancement component caused by the Brownian motion was crystal clearly observed. Likewise to the expected dose dependence, the highest thermal conductivity enhancement seem observed on the nanofluids for which the Ag nanoparticles exhibited a crystallographic texture (in this case (220) texture). Yet this was observed in both Ag-H_2_O and Ag-C_2_H_6_O_2_, it is premature to conclude on such an aspect. As a foresight, the investigation of the decrease of the thermal conductivity at higher doses would be investigated further.

## Supplementary Information

Supplementary Information.

## Data Availability

In line with the journal’s policy and regulations, the data will be available upon request addressed to the corresponding author (Maazam@unisa.ac.za, Maaza@tlabs.ac.za).
