# Supplementary material for: A novel approach for engineering efficient nanofluids by radiolysis
Source: Sci Rep. 2022 Jun 24;12:10767. doi: 10.1038/s41598-022-14540-z (PMC9232626; doi:10.1038/s41598-022-14540-z)
Supplement: Supplementary file 1 — Supplementary Information. [file 41598_2022_14540_MOESM1_ESM.pdf]

ADDITIONAL INFORMATION

A novel approach for engineering efficient nanofluids by radiolysis

1. Experimental & Methodologies

1.1. Radiolysis synthesis details:

For the samples preparation, silver nitrate of high chemical purity (AgNO3, Merck, Germany) was used as the starting source of silver for the radiolytic-induced reduction of Ag+ to Ag0. De-ionized H_2O & standard purity C2H6O2 solutions containing 40 mM $AgNO_3$ were prepared. After deaeration by bubbling with nitrogen gas, the solutions were irradiated by gamma rays emitted by a standard panoramic 1MCi ^{60}Co source in a regular configuration as schematically represented in Fig.2.a & Fig.S.1. The standard irradiations were carried out at various gamma irradiation doses of D_1= 0.95, D_2=1.25, D_3= 1.54, D_4= 1.80 & D_5= 2.45 10^3 Gray and at a dose rate of 10.0 10^3 Gy/h for each of the prepared 40 mM $AgNO_3$ in H_2O & in $C_2H_6O_2$ solutions. These doses were chosen based on the published literature (32-34). More precisely, such a set of doses allows the synthesis of homogeneous nano-scaled Ag colloidal suspensions (32-34), with a relative stability of months.

The technical details of the ^{60}Co source can be found from the supplier (Eckert & Ziegler*)*

In a summarized way, 4 phases are considered:

Phase-1: Preparation of the De-ionized H_2O & standard purity $C_2H_6O_2$ solutions containing 40 mM $AgNO_3$.,

Phase-2: Deaeration by bubbling with nitrogen gas the various solution before any γ-irradiation,

Phase-3: The solutions are irradiated by γ-radiations emitted by a standard panoramic 1MCi ^{60}Co source in a regular configuration as schematically represented in Fig.S.1.The irradiation is carried out at a dose rate of 10.0 10^3 Gy/h while the sample container is rotating (~40 rpm) in view of an homogeneous irradiation. The time of irradiation is varied from 5.7 min,7.5 min,9.24 min, 10.8 min, 14.7 min. These irradiations correspond to γ-radiations doses of D_1= 0.95, D_2=1.25, D_3= 1.54, D_4= 1.80 & D_5= 2.45 10^3 Gray and at a dose rate of 10.0 10^3 Gy/h.

Phase-4: Following the various morphological, crystallographic & optical investigations, the thermal conductivity were carried using the hot wire methodologies in view of its efficiency and simplicity in addition to its significant reliability.

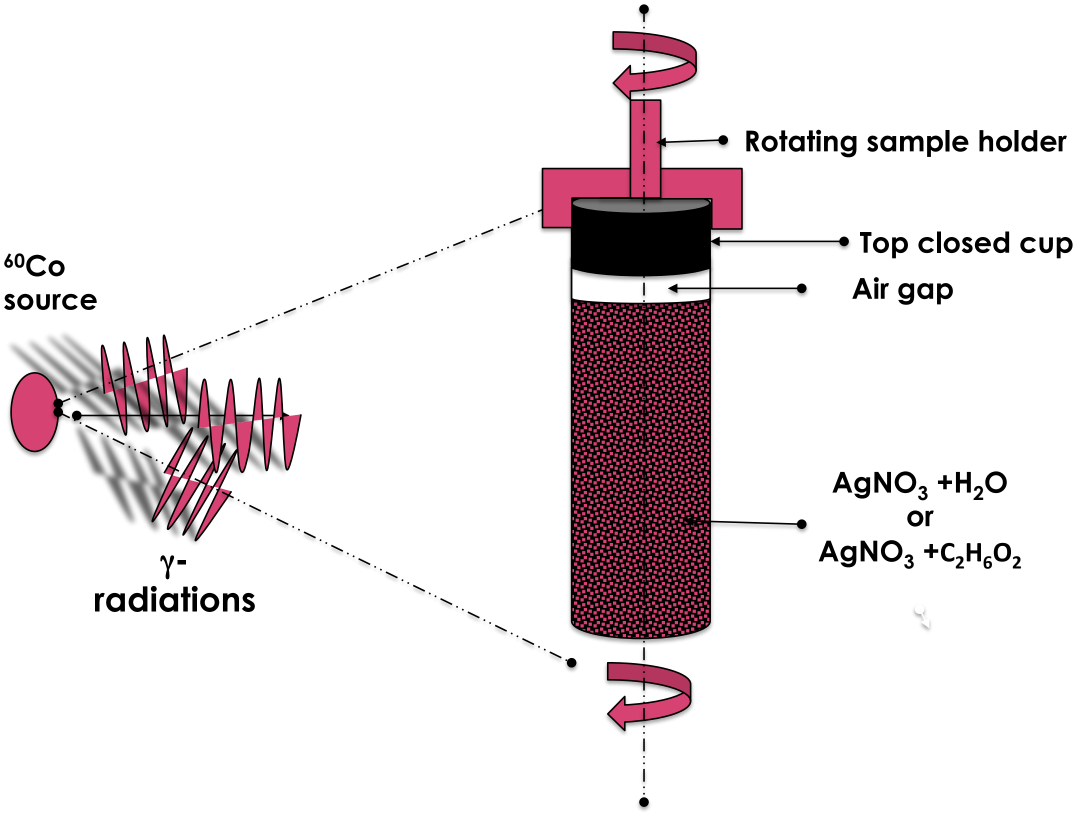

Fig.S.1: Schematic representation of the synthesis of the nanofluids by radiolysis.

1.2. Electron microscopy investigations:

The imaging & electron diffraction of Fig.3, Fig.4 & Fig.5 were collected using a JEM-2100 Plus electron microscopy. This latter is a multipurpose 200kV LaB6 TEM that provides solutions for a wide range of applications from materials science to medical/biological studies. It allows a relatively line probe analysis and nano beam diffraction with a probe size <1nm, and an effortless atomic/lattice imaging capabilities < 0.14nm with Point-Point resolution guaranteed as low as 0.194nm with the UHR objective lens, in addition to crystallographic electron diffraction option. Also, the unit allows tomography-capabilities with a large range of tilt (¬+80°) imaging. The technical details of the JEM-2100 Plus can be found from the supplier (JEOL)**

1.3. Thermal conductivity investigations:

The thermal conductivity of the engineered nanofluids was investigated by the standard transient hot-wire technique (25) within the temperature range of 25-50°C. As established, the

accuracy of this hot wire approach (order +0.2%) and precision (order 0.02%) have been obtained as a result of the application of modern electronic instruments of a superior quality (25, 44,52). Fig.S1 reports the schematic configuration for the measurements of the thermal conductivity using the hot wire approach. The comprehensive theoretical of such approach is presented elegantly by Healy et al (25). The full description of the technical aspect is summarized in Alvarado et al (52).

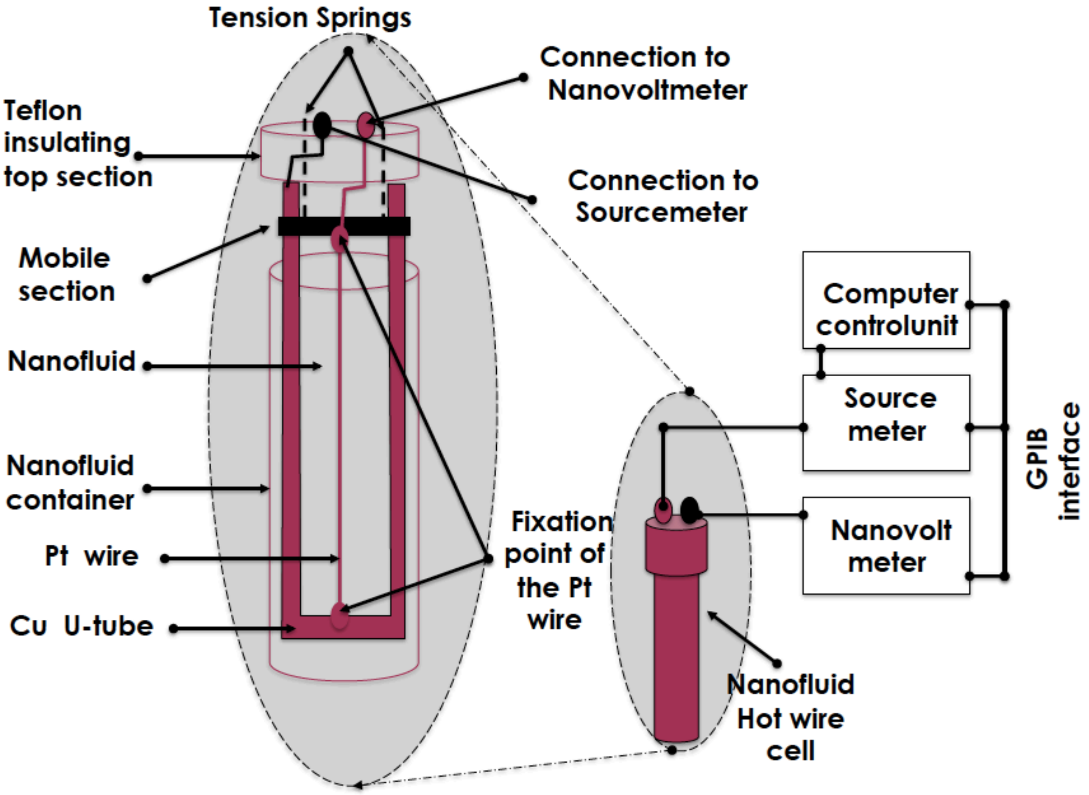

Fig.S.2: Schematic description of the standard Hot Wire set up for the thermal conductivity measurements.

* https://www.ezag.com/fileadmin/ezag/user-uploads/pdf/isotope/5_industrial_sources.pdf

** https://www.azom.com/equipment-details.aspx?EquipID=8035
